# Supplementary figures and images for: Decoding the chemical language of Suillus fungi: genome mining and untargeted metabolomics uncover terpene chemical diversity
Source: mSystems. 2024 Mar 12;9(4):e01225-23. doi: 10.1128/msystems.01225-23 (PMC11019867; doi:10.1128/msystems.01225-23)

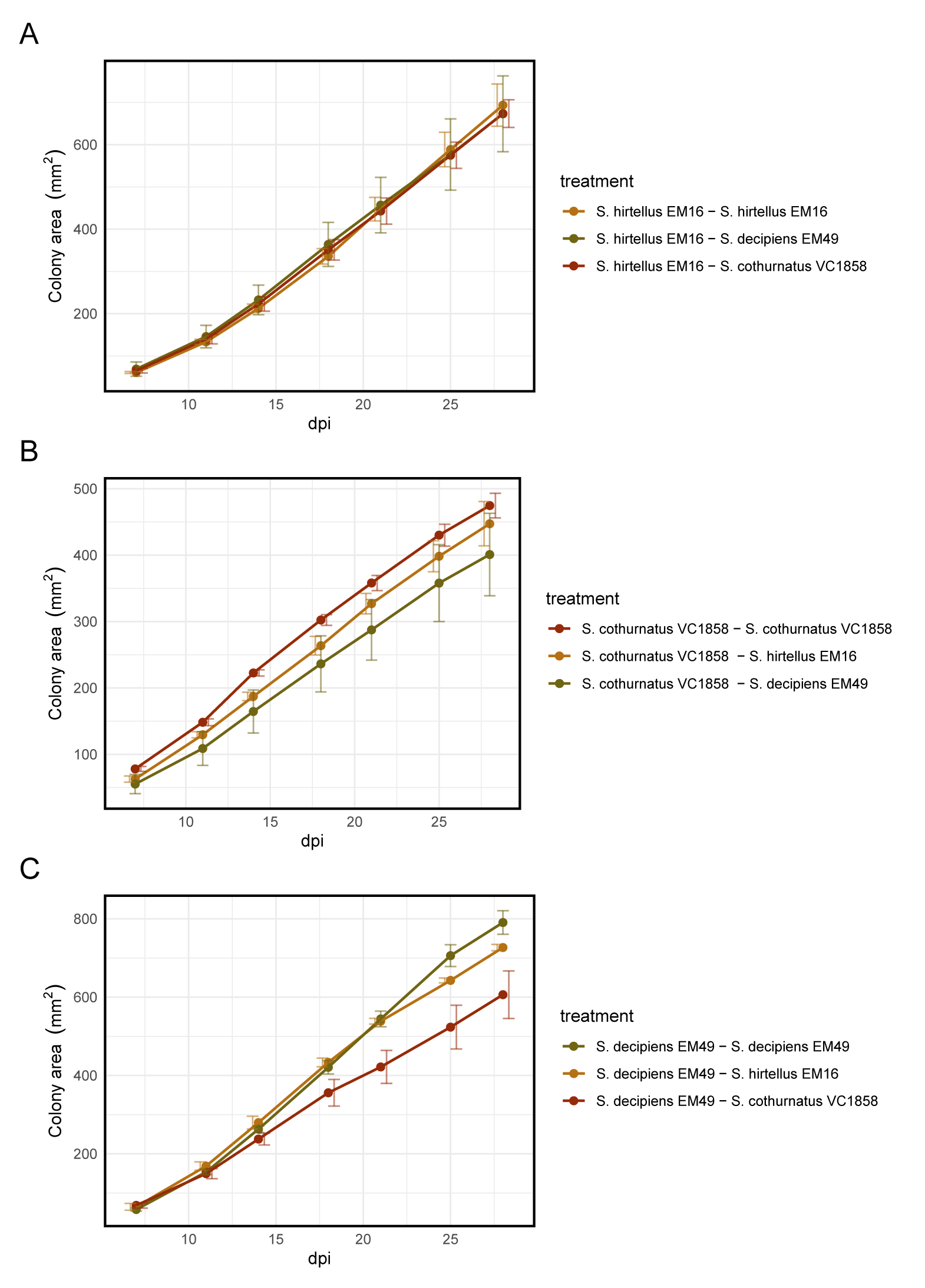

Supplement: Figure S1 — Growth differences observed for Suillus species grown in intra- and inter-species pairings. [file msystems.01225-23-s0001.tif]

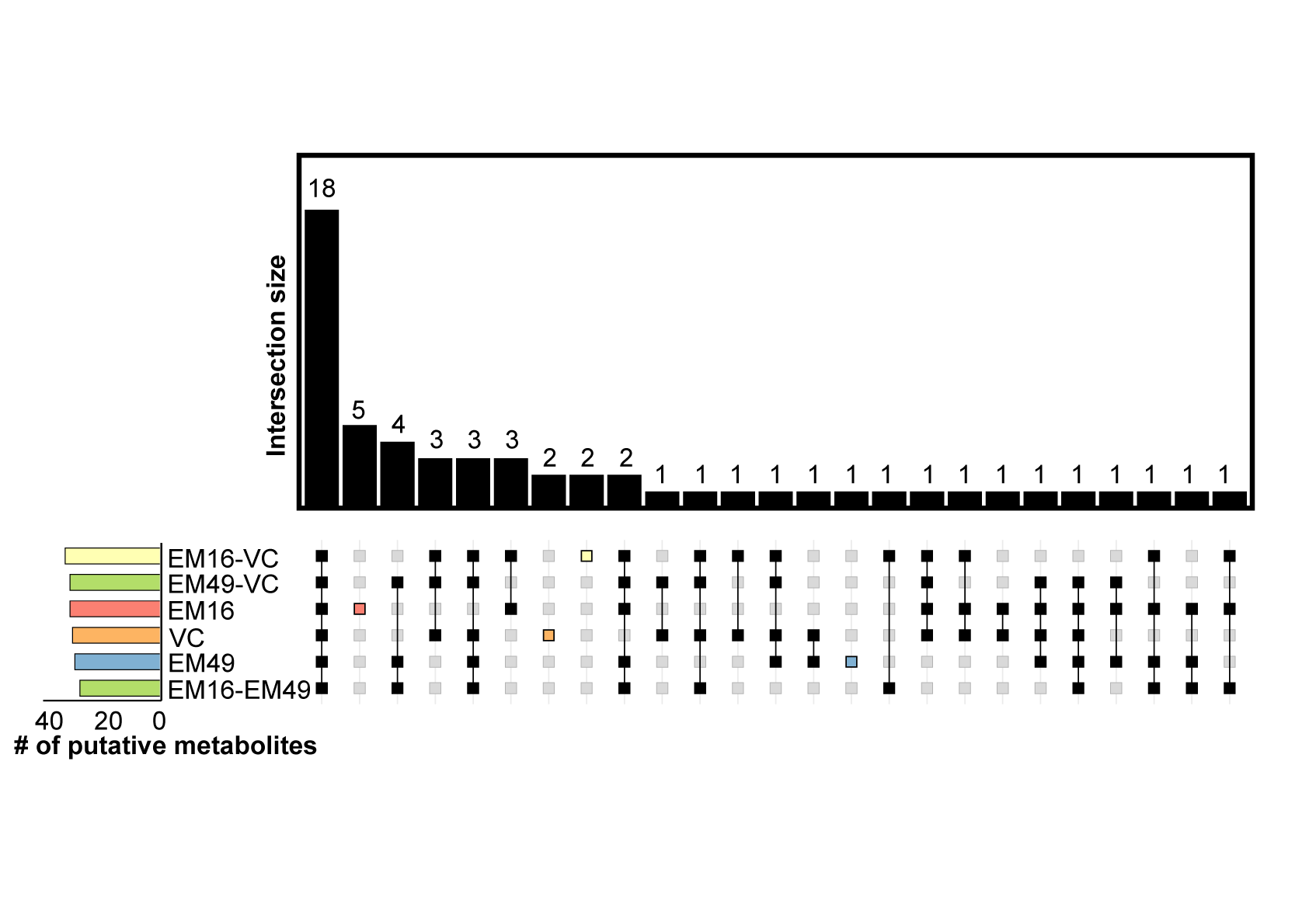

Supplement: Figure S2 — The chemical diversity for the putatively identified metabolites in the aqueous fraction. [file msystems.01225-23-s0002.tif]

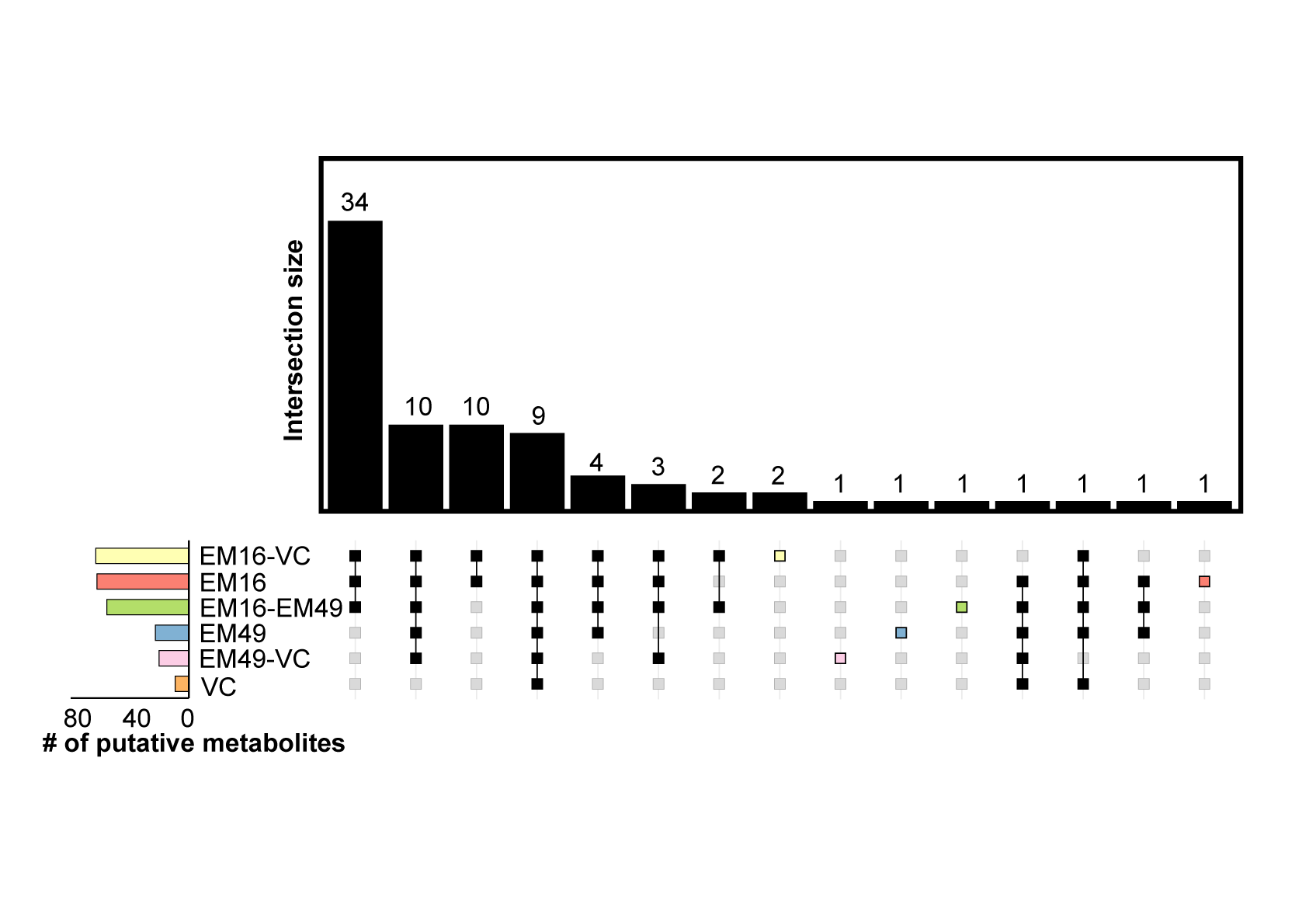

Supplement: Figure S3 — The chemical diversity for the putatively identified metabolites in the organic fraction. [file msystems.01225-23-s0003.tif]

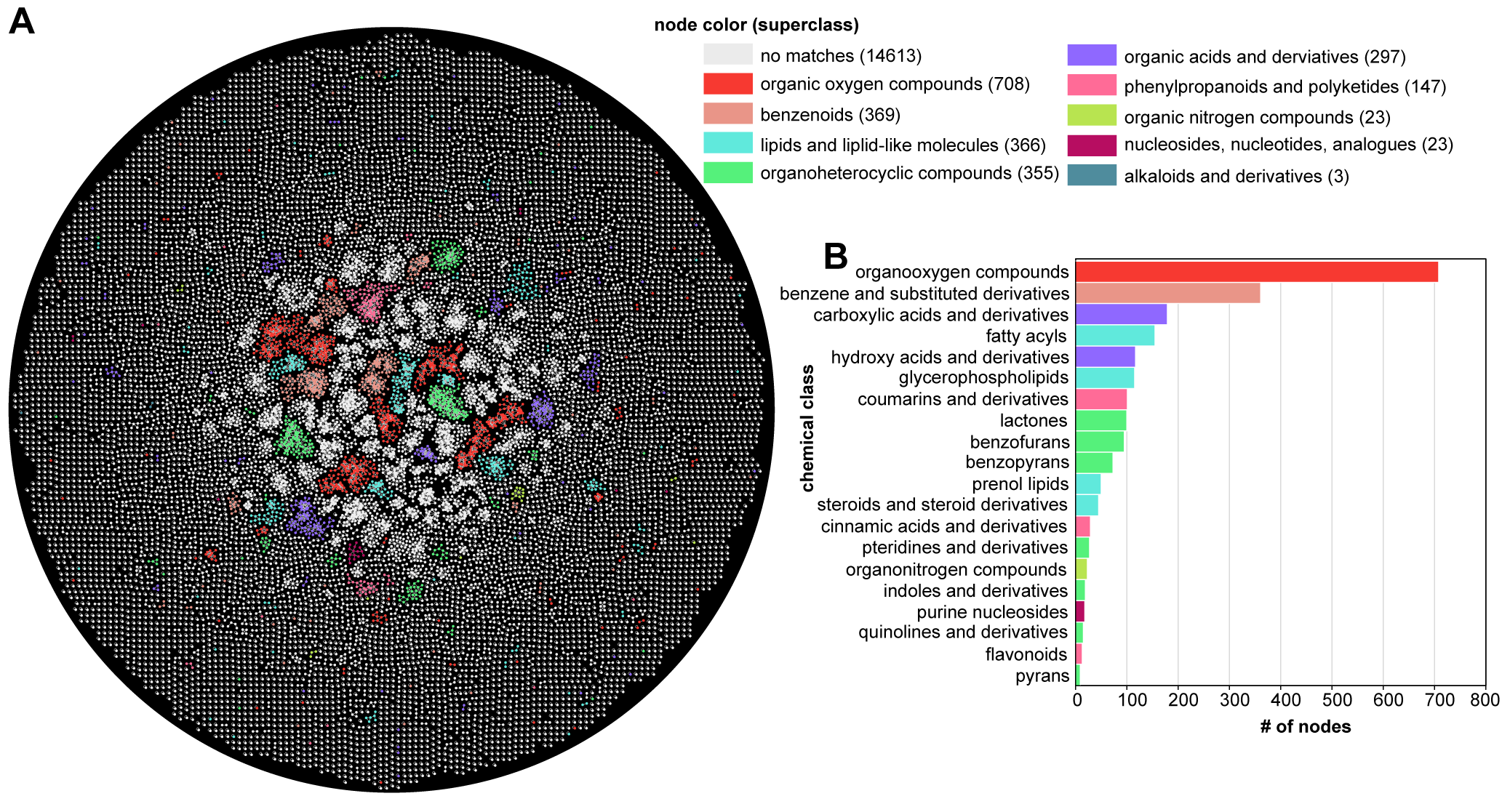

Supplement: Figure S4 — Classical molecular network uncovered predominant annotated and unannotated MS/MS spectra. [file msystems.01225-23-s0004.tif]
