# Supplementary material for: Decoding the chemical language of Suillus fungi: genome mining and untargeted metabolomics uncover terpene chemical diversity
Source: mSystems. 2024 Mar 12;9(4):e01225-23. doi: 10.1128/msystems.01225-23 (PMC11019867; doi:10.1128/msystems.01225-23)
Supplement: Supplemental File — Additional details provided for LC-MS methods and supplemental legends. [file msystems.01225-23-s0005.docx]

# **Additional Materials and Methods**

## *Detailed listing of LC-MS parameters:*

Composition for solvent A 95 % water, 5 % acetonitrile, 0.1% formic acid

Composition for solvent B 70% acetonitrile, 30 % water, 0.1% formic acid

LC Vanquish method:

0.000 [min]

Flow: 0.150 [ml/min]

%B: 0.0 [%]

15.000 [min]

Flow: 0.150 [ml/min]

%B: 100.0 [%]

16.000 [min]

Flow: 0.150 [ml/min]

%B: 100.0 [%]

18.000 [min]

Flow: 0.150 [ml/min]

%B: 0.0 [%]

30.000 [min]

Flow: 0.150 [ml/min]

%B: 0.0 [%]

MS Q Exactive plus method:

*Full MS:*

Microscans: 1

Resolution: 70,000

AGC target: 3e6

Maximum IT: 100 ms

Number of scan ranges: 1

Scan range: 135 to 2000 m/z

Spectrum data type: Profile

*dd-MS² / dd-SIM:*

Microscans: 1

Resolution: 17,500

AGC target: 1e6

Maximum IT: 50 ms

Loop count: 5

MSX count: 1

TopN: 5

Isolation window: 2.0 m/z

Isolation offset: 0.5 m/z

Scan range: 200 to 2000 m/z

Fixed first mass: ―

(N)CE / stepped (N)CE: nce: 10, 20, 40

Spectrum data type: Profile

*dd Settings:*

Minimum AGC target: 8.00e3

Intensity threshold: 1.6e5

Apex trigger: ―

Charge exclusion: unassigned, >8

Peptide match: Off

Exclude isotopes: on

Dynamic exclusion: 10.0 s

If idle: do not pick others

**Supplemental File Legends:**

**Figure S1:** Growth differences observed for *Suillus* species grown in intra- and inter- species pairings. (A, B, C) showing increase in colony area (mm^2^) from Day 0 until Day 28 when agar plugs were taken for exometabolomic analysis for *S. hirtellus* EM16, *S. cothurnatus* VC1858 and *S. decipiens* EM49 respectively when they are grown in either monoculture or in coculture with other species. As can be seen in the figure there is only a marginal growth difference when grown either as a inter species pair or in an intra species monoculture.

**Figure S2:** The chemical diversity for the putatively identified metabolites in the aqueous fraction. The UpSet plot shows the number of chemical classes observed in each sample as well as the total overlap in chemical classes between each sample.

**Figure S3:** The chemical diversity for the putatively identified metabolites in the organic fraction. The UpSet plot shows the number of chemical classes observed in each sample as well as the total overlap in chemical classes between each sample.

**Figure S4:** Classical molecular network uncovered predominant annotated and unannotated MS/MS spectra. (A) The GNPS-derived molecular network was visualized by the Cytoscape software. Each node represents a MS/MS spectrum from this study. Nodes colored white represent unannotated MS/MS spectrum and colored nodes represent a MS/MS spectrum associated with a putative metabolite annotation with a relatively high spectral similarity score (correlation >0.7). Nodes with putative annotations were annotated by the MolNetEnhancer workflow in GNPS to illustrate the different superclass annotations. (B) A bar plot shows the number of metabolites belonging to a chemical class reported by the MolNetEnhancer workflow in GNPS.

**Table S1:** Verbose data matrix of identified compound features generated from the software Compound Discoverer v3.3.

**Table S2:** Subset of Compound Discoverer verbose data matrix filtered to compound features that matched with confidence against mzCloud and/or NIST2020 spectral libraries.

**Table S3:** A non-redundant list of spectral matched compound features filtered to those with abundances significantly greater than media blank (Log2FC >2 and adj. p-value < 0.05 ).

**Table S4:** ClassyFire chemical classifications are listed for metabolites identified from the Compound Discoverer workflow.

**Table S5:** A data matrix of putative metabolites identified by the GNPS classical molecular network workflow.
